# The Safety Conversation: developing a trustwide safety conference at CNWL during a pandemic

**DOI:** 10.1192/bjo.2021.382

**Published:** 2021-06-18

**Authors:** Emily Duncan, Simon Edwards, Alison Butler, Cornelius Kelly

**Affiliations:** Central and Northwest London NHS Trust

## Abstract

**Aims:**

The COVID pandemic has had both a massive impact on clinical service delivery and the way that training and education is provided. CNWL is a large NHS provider and has approximately 7000 staff working across 150 locations, providing mental health and community health services. In response to the need to share learning across the organisation, a trustwide “Safety Conversation Day” took place to spotlight the work being done to promote safety and to act as a platform to share ideas and learning across the trust. This was the first ever virtual conference organised by the trust.

**Method:**

The one-day conference included virtual posters and an all-day open access virtual conversation delivered via zoom. The day was divided into 6 safety themes: Safety tools; Safer Environments; Supporting and Involving Staff; Safer use of Medicines; See Think Act and Relational Security; and Prevention is Better than Cure. Frontline staff delivered 5-6 short presentations each hour highlighting new ways of working, quality improvement, local research etc.

Staff were also encouraged to submit posters for the event, with webinars held on how to write a poster held prior to the safety conversation to promote engagement. Prizes were awarded for best posters in the different categories.

A mentimeter survey was running throughout the day to get feedback from participants.

**Result:**

This was the largest event of this kind held by the trust. 430 unique viewers logged in during the day to watch the presentations.

Feedback was very positive on the mentimeter survey. 3 questions were asked on a likert scale of: Strongly Disagree – Strongly Agree (rated out of 5):

- ‘I found the posters really useful': 4.5/5
- ‘I found the presentations very useful': 4.6/5
- ‘I will share what I've learnt about safety': 4.6/5

Open space questions and word cloud responses also highlighted qualitative feedback with most frequent responses including ‘inspiring', ‘interesting and ‘stimulating’.

174 posters were presented with good representation from all services and staff groups across the trust (18 on safer use of medicines, 15 on co-production, 52 on quality improvement, 50 on COVID and non-COVID safety, 16 on use of technology, 23 on supporting and involving staff). These posters have since been downloaded 4062 times.

**Conclusion:**

The first CNWL safety conference proved an excellent opportunity to celebrate achievements in patient safety in a very difficult year. It was very well-received and well-attended by staff, promoting maximal learning across the organisation.

